# Immunohistological Localization of BMP-2, BMP-7, and Their Receptors in Knee Joints with Focal Cartilage Lesions

**DOI:** 10.1100/2012/467892

**Published:** 2012-01-03

**Authors:** Hagen Schmal, Alexander T. Mehlhorn, Ingo H. Pilz, David Dovi-Akue, Christina Kirchhoff, Norbert P. Südkamp, Ulrike Gerlach, Christian Lohrmann, Philipp Niemeyer

**Affiliations:** ^1^Department of Orthopaedic Surgery, University Medical Center Freiburg, Hugstetter Street 55, 79106 Freiburg, Germany; ^2^Institute of Pathology, University of Medical Center Freiburg, Breisacher Street 115a, 79106 Freiburg, Germany; ^3^Department of Radiology, University of Medical Center Freiburg, Hugstetter Street 55, 79106 Freiburg, Germany

## Abstract

*Introduction*. Although it is well known that BMP-2 and BMP-7 play significant roles in cartilage metabolism, data about intra-articular expression and localization of these proteins and their receptors in humans are rare. *Methods*. Biopsies of synovia and debrided cartilage were taken in patients undergoing autologous chondrocyte implantation. Expression of BMP-2, BMP-7, and their receptors BMPR-1A, BMPR-1B and BMPR-2 were semiquantitatively evaluated by immunohistological staining. *Results*. BMP-7 was equally highly expressed in all cartilage and synovial biopsies. Increased levels of BMPR-1A, but not of BMPR-1B, and BMPR-2, were found in all synovial and 47% of all cartilage samples (*P* = 0.002). BMP-2 was positively scored in 47% of all cartilage and 40% of all synovial specimens. Defect size, KOSS, Henderson or Kellgren-Lawrence score did not statistically significant correlate with the expression of the analyzed proteins or Mankin and Pritzker scores. Duration of symptoms and localization of lesions were associated with KOSS (*P* < 0.02), but there was no influence of these parameters on protein expression. *Conclusions*. BMP-2, BMP-7, and BMPR-1A were expressed in cartilage and synovia of knees with focal cartilage lesions. Although defect localization and duration of symptoms decisively influence KOSS, there was no associated alteration of protein expression observed.

## 1. Introduction

BMPs belong to the transforming growth factor-beta (TGF-*β*) superfamily, consisting of the different forms of TGF-*β*, and other growth differentiation or neurotrophic factors [1]. Effects of BMP-2 and BMP-7 are numerous, for example, they play a significant role in skeletal development [2] and are potent inducers of bone formation [3]. Therefore, several years ago both proteins gained their registration approval as pharmaceuticals to treat delayed fracture healing or spinal fusions. BMP-2 is able to promote chondrogenesis in human mesenchymal stem cells [4], in which the TGF-*β*-driven tendency to develop cartilage hypertrophy is partially inhibited. The ability of BMP-2 to induce chondrogenesis has been demonstrated in different experimental set-ups [5]. BMP-2 coating of scaffolds resulted in mature cartilage formation using either mesenchymal stem cells or amplified chondrocytes [6]. Furthermore, mechanical stress was found to upregulate BMP-2 as well as BMP-2 signaling in human cartilage explants [7], indicating a role for BMP-2 in natural cartilage reparative processes. BMP-7 is also known as osteogenic protein-1 (OP-1) and exhibits characteristics as an anabolic factor in cartilage metabolism. BMP-7 was able to enhance synthesis of extracellular matrix components and to promote cartilage repair. This could be shown for both articular and disc cartilage applications [8]. BMP-7 and its receptors have been immunohistologically identified in rabbit articular cartilage and bone, suggesting a possible role in cartilage and bone homeostasis [9]. BMP-7 was also detected in human articular chondrocytes showing differential regulation in normal and osteoarthritic cartilage [10]. BMP effects are mediated by type 1 and type 2 receptors, which act as intrinsic serine- and threonine kinases. BMP receptors usually form active dimers and signal via the downstream molecules Smad1, 5, and 8. Three type 1 receptors (BMPR-1A or ALK-3, ActR-1A or ALK-2, and BMPR-1B or ALK-6) and three type 2 receptors (BMPR-2, ActR-2, and ActR-2B) are discriminated, but only BMPR-1A, 1B, and 2 are specific to BMPs [11]. The signaling is highly regulated at different molecular levels, for example, Noggin blocks BMP signaling and Smad6 prevents activation of Smad1, 5, and 8. Therefore, overexpression of noggin in mature osteoblasts causes osteoporosis in mice [12] and overexpression of Smad6 in chondrocytes causes delays in chondrocyte differentiation and maturation [13]. Smad4 serves as an exclusive coactivating smad that elicits most of the transcription responses invoked by the TGF-*β* superfamily members, deletion of Smad4 also leads to defective chondrocyte maturation [14]. Recently, the role for BMP-2 in surgically induced cartilage repair was emphasized, since it has been shown that synovial expression correlated with the clinical outcome after 1 year [15]. Although other proteins with known roles in cartilage metabolism as bFGF, IGF-I, or BMP-7 were present in lavage fluids of knee joints [16], neither of these cytokines was statistically significant associated with IKDC score following 1 year. Data of this study was based on analyses of perioperative lavages of knees, but it remained unclear where BMPs are located and what clinical parameters may influence BMP-2 or BMP-7 expression. This study was initiated in order to answer this question and to further clarify localizations of the receptors BMPR-1A, BMPR-1B, and BMPR-2. Several radiological scores have been established in order to define progress of osteoarthritis (OA) as the Kellgren-Lawrence score [17] for conventional radiographies or the KOSS score for magnetic resonance imaging (MRI) [18]. Since we hypothesized a correlation of BMP expression and their receptors with OA, a possible association between the mentioned radiographic scores and immunohistologically determined BMP expression was examined.

## 2. Material and Methods

### 2.1. Study Design

15 patients were enrolled in a prospective clinical trial between 01.01.2010 and 30.06.2010. Selection of patients followed the criteria as defined beneath.

Inclusion criteria were the performance of an autologous chondrocyte implantation (ACI) of the knee joint because of full thickness cartilage lesions graded III and IV according to ICRS classification [19] of various size, agreement to participate in the study, age >17 years, and <66 years (as recommended [20]).

Exclusion criteria were alcohol or drug abuse, mental retardation with incapability to complete the necessary self-reports, joint effusion >30 mL, persistent knee instability, and infection. 

The study was approved by the ethical board of the University of Freiburg (AN-EK-FRBRG-64/10, study number DRKS00000487). An informed consent was obtained from every subject included in the study.

Besides histological data, the following parameters were collected: epidemiological characteristics, defect size (cm^2^), duration of symptoms (months), Knee Osteoarthritis Scoring System (KOSS) for evaluation of OA progress in preoperative magnetic resonance images (MRI) as described in [18], Henderson score for evaluation of subchondral edema as described in [21], Kellgren-Lawrence score as described in [22].

### 2.2. Specimen Collection

The ACI surgical technique has been well defined in numerous publications [20, 23, 24]. Implantation consists of arthrotomy and defect preparation without affecting the subchondral bone layer. This includes debridement of the repair or defect cartilage zone, gaining sharp edges of healthy adjacent cartilage in order to realize a proper containment. The removed cartilage debris was collected and kept in formalin for later histological analysis. Furthermore, a biopsy of synovia out of the arthrotomy region was taken and separately preserved in formalin.

### 2.3. Grading of Cartilage Lesion

The amount of chondral damage was graded from 0 to 4 based on the ICRS classification [19]. Grade 0 represents normal articular cartilage and grade I shows superficial lesions as soft indentation and/or superficial fissures and cracks. A grade II defect is a partial-thickness defect; it features lesions extending down to less than 50% of cartilage depth. With grade III defects, there are cartilage defects extending down to more than 50% of cartilage depth as well as down to the calcified layer, and down to but not through the subchondral bone. Blisters are included in this grade. In grade IV injuries, the subchondral bone is involved. Decision about grading of the cartilage lesion was intraoperatively done, when the surgeon debrided the defect zone. 

### 2.4. Histology

Specimen were fixed in 4% paraformaldehyde and dehydrated in graded series of ethanol. Samples were embedded in paraffin and cut (3 *μ*m) on a Leica RM 2255. Sections were incubated in alcian blue solution or hematoxylin and subsequently eosin as previously described [25]. The slides were washed, dried at room temperature, and coverslips were mounted with Roti-Histokitt II mounting media.

### 2.5. Immunohistology

All used antibodies (AB) were purified IgG isotype AB and tested for immunohistological applications with human epitopes by all manufacturers. As control tissue small intestine, kidney, placenta, or cartilage was used. Stainings were done using an Autostainer Plus S3400 (DAKO, Hamburg, Germany) with the appropriate controls for each run. Sections were fixed in 4% paraformaldehyde, dehydrated, embedded in paraffin, and cut in 2 *μ*m slices. Before incubation with primary antibodies, antigens were unmasked using the indicated method (Table 1). For immunostaining a Dako REAL Detection System (Alkaline Phosphatase/RED, Rabbit/Mouse, K5005) was used according to the manufacturer's instructions (DAKO, Hamburg, Germany). Briefly, primary antibodies were applied using the indicated dilution (Table 1). Antibody diluent (ZUC 025-500, Zytomed Systems, Berlin, Germany) was used for all preparations. After washing with Dako Wash Buffer (S 3006), REAL biotinylated secondary antibodies (AB2) were applied at a 1 : 100 dilution. After washing, Dako REAL Streptavidin Alkaline Phosphatase was applied followed by the RED chromogens 1–3. In order to inhibit endogenous alkaline phosphatase activity, Dako REAL Levamisole was added to the substrate. For controls, the primary antibody was replaced by either normal serum, the secondary antibody was applied alone, or control tissue was used. Finally, slides were counterstained with haematoxylin.

### 2.6. Evaluation of Slides and Radiographs

Immunostainings or radiographs were assessed by two independent referees. In case of disagreement, a consensus evaluation was found with support of a third referee. Intensity (0–4) of staining and percentage of stained cells (1–3) were estimated and a summary score was calculated. Since staining of the different regions partially showed varying intensity, areas with the least strength were regularly used for scoring. General histological stainings were used to determine Mankin score [26], Pritzker (OARSI) score [27], and Krenn score [28]. The KOSS score was used for quantitative evaluation of OA signs in MRI [18], the Kellgren-Lawrence score [17] for conventional radiographies.

### 2.7. Statistics

All values were expressed as mean ± standard deviation. Data sets were compared with the rank sum *U*-Test (Mann-Whitney). Significance of correlations was determined by calculating the Spearmen (Rho) coefficient. Categorical data are presented as absolute frequency. Data (incidences) were arranged in cross tables, and statistical significance of differences calculated using the 2-tailed Fisher exact test. Statistical significance was defined when *P* < 0.05.

## 3. Results

### 3.1. Study Parameters

The average age of all included patients (15) was 33.12 ± 11.11 years, the gender distribution was 9/6 male/female individuals. The retropatellar region was affected 6 times, the medial femoral condyle 8 times. Once the trochlea was affected in combination with the lateral femoral condyle. The ACI was performed alone in 5 cases; otherwise transplantation was supplemented by correction of leg axis (4), soft tissue balancing measures for the patella (4), replacement of the anterior cruciate ligament (1), or bone grafting (1). The average defect size was 3.83 ± 1.48 cm^2^. In only 3 cases ACI was the first operation of the knee, in the other cases ACI was preceded 7 times by cartilage regenerating surgery as microfracturing, 4 times by partial meniscus resections, once by a correction of leg axis, once by patella balancing, and once by removal of free joint bodies (multiple nominations per patient possible, 14 operational measures in 12 patients). The preoperative ICRS score was 3 in 7 cases and 4 in 8 cases. An MRI and a conventional X-ray of the affected knee made within 180 days before the operation were available in all patients. The mean KOSS was 7.13 ± 2.13, the mean Kellgren-Lawrence score was 0.93 ± 0.70. Average preoperative duration of complains was 51.73 ± 33.95 months [6-120].

### 3.2. Immunohistological Analysis of BMP-2, BMP-7, BMPR-1A, BMPR-1B, and BMPR 2 Expression

**Figure 1:**
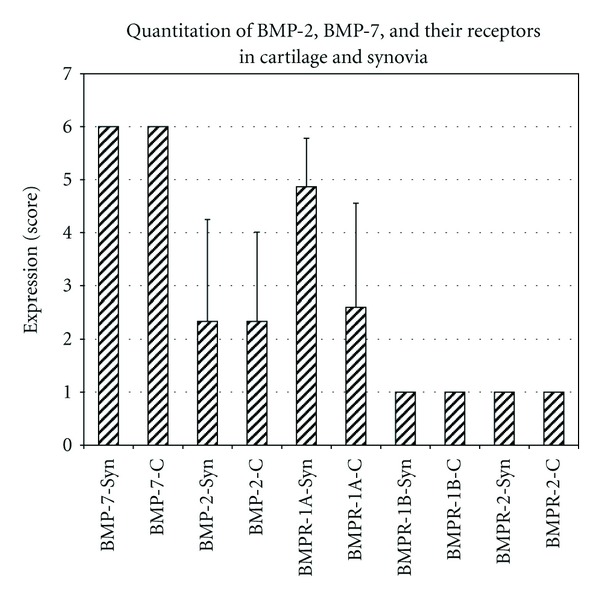
Quantitation of BMP-2, BMP-7, and their receptors in cartilage and synovia.

**Figure 2:**
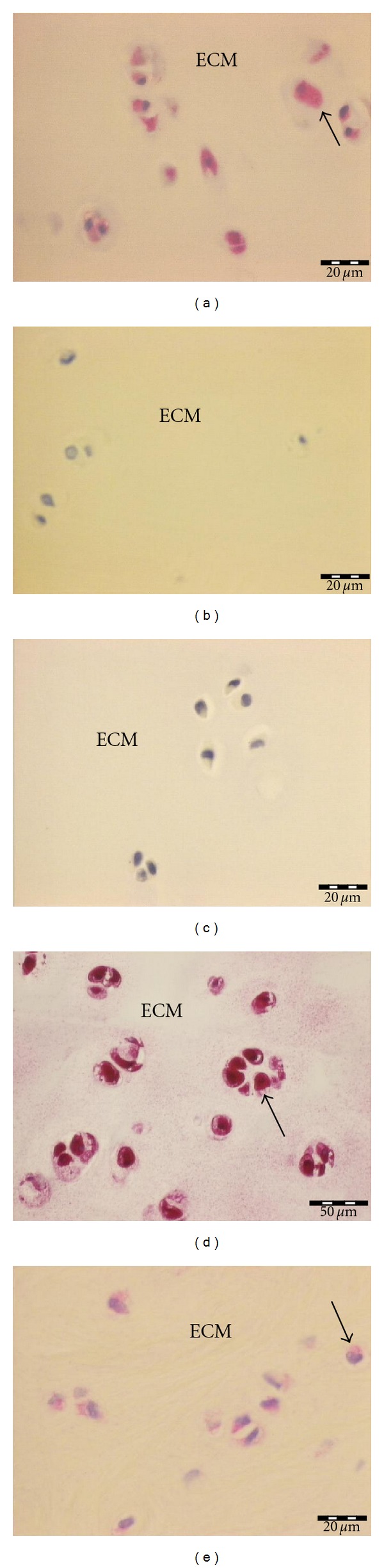
Immunostaining of cartilage, (a) BMPR-1A (summary score 6), (b) BMPR-1B (summary score 1), (c) BMPR-2 (summary score 1), (d) BMP-7 (summary score 6), (e) BMP-2 (summary score 4).

**Figure 3:**
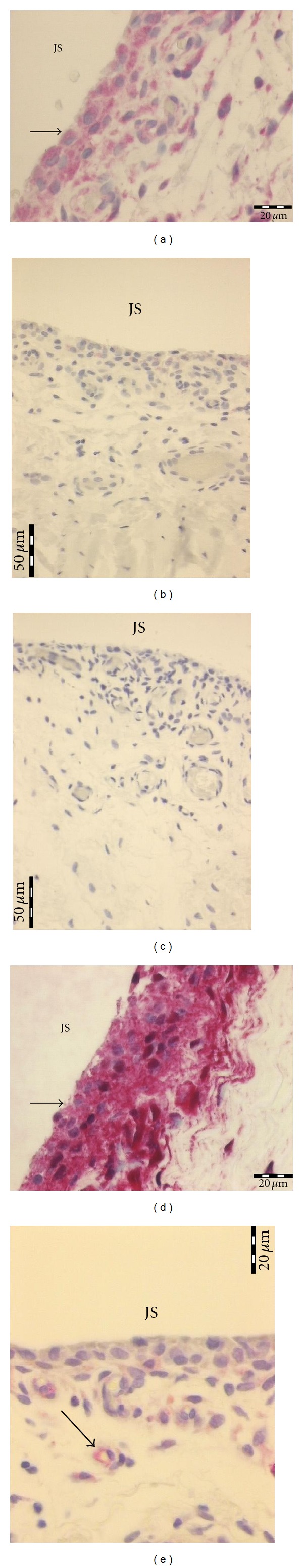
Immunostaining of synovia, positive staining (arrows) is indicated by the red color mainly located around the nucleus of chondrocytes, (a) BMPR-1A (summary score 6), (b) BMPR-1B (summary score 1), (c) BMPR-2 (summary score 1), (d) BMP-7 (summary score 6), (e) BMP-2 (summary score 2).

BMP-7 was highly expressed in all cartilage and synovial biopsies, sum of scores reached 90 points, the maximum of possible points. Positive staining of BMPR-1B and BMPR-2 was only sporadically found without an increase of the semiquantitative scores over background levels (sum score 15 points, the minimum of possible points, in synovial and cartilage for both proteins). BMP-2 was positively scored in 47% of all cartilage (sum score 35 points) and 40% of all synovial specimens (sum score 35 points). Increased levels of BMPR-1A were found in all synovial samples (sum score 73 points), but only in 47% of all cartilage biopsies (sum score 39 points). BMPR-1A was the only investigated protein with statistically significant different expression in cartilage and synovia (*P* = 0.002). Expression of BMP-7 was statistically significant higher in synovia compared to BMP-2 (*P* < 0.001), BMPR-1A (*P* = 0.0002), BMPR-1B (*P* < 0.001), and BMPR-2 (*P* < 0.001). Expression of BMP-7 was statistically significant higher in cartilage compared to BMP-2 (*P* < 0.001), BMPR-1A (*P* < 0.001), BMPR-1B (*P* < 0.001), and BMPR-2 (*P* < 0.001). Expression of BMPR-1A in synovia (*P* < 0.0001) and cartilage (*P* = 0.003) was statistically higher compared to BMPR-1B and BMPR-2. BMPR-1A-expression in synovia scored higher than BMP-2 (*P* = 0.0015), but was not different in cartilage (*P* = 0.7863). An overview about the summary scores is given in Figure 1.  Figure 2 shows representative immunohistological stainings of cartilage, Figure 3 of synovia.

### 3.3. Correlation Analysis of Radiographic Scores, Clinical, and Histological Parameters

The parameters defect size, KOSS score (grading of OA in MRI), Henderson score (subchondral edema), Kellgren-Lawrence score (grading of OA in conventional radiographs), duration of symptoms (months), and Krenn score (grading of synovitis) were correlated with each other in order to elucidate possible associations between clinical symptoms and morphological changes visible in different imaging techniques. Defect size was statistically significant associated with KOSS score (Rho = 0.6259, *P* = 0.0063) and Kellgren-Lawrence score (Rho = 0.4705, *P* = 0.0384). KOSS score was further highly statistically significant associated with Henderson score (Rho = 0.6133, *P* = 0.0075), which is not surprising, because grading of edema is part of the KOSS score. Interestingly, KOSS score statistically significant correlated with duration of symptoms (Rho = 0.6272, *P* = 0.0062), that appears the only association between imaging and symptoms. Data are summarized in Table 2. Furthermore, defect sizes, KOSS score, Henderson and Kellgren-Lawrence score were analyzed for their possible association with the expression of BMPR-1A and BMP-2. There was no statistically significant correlation of these parameters with the expression of the analyzed proteins or Mankin and Pritzker score. BMP-7, BMPR-1B, and BMPR-2 did not show alterations between different patients and were excluded for this analysis.

### 3.4. Analysis of the Influence of Duration of Symptoms

Correlation analysis (Table 2) revealed that duration of symptoms appeared as a critical factor. Therefore, the possible influence on the other study parameters was evaluated. At first the critical time period for the significance was calculated. When dividing the individuals in two groups with ≤48 months and >48 months of complains, the number of patients in each group was almost equal and mean KOSS scores statistically significant differed (*P* = 0.0202). All other parameters including OA and immunohistological scores did not show any statistical significant difference as summarized in Table 3. Only the summary scores of BMPR-1A and BMP-2 were integrated in the analysis, because scores of BMP-7, BMPR-1B, and BMPR-2 did not show alterations between different patients.

### 3.5. Analysis of the Influence of Localizations of Chondromalacia

Since localizations of chondromalacia (CM) has previously been described as a critical factor for long-term prognosis following treatment of cartilage defects [29], this parameter was evaluated in this study with regard to a possible different expression of BMPR-1A and BMP-2. Because scores of BMP-7, BMPR-1B, and BMPR-2 did not show alterations between different patients these scores were excluded. 6 patients with retropatellar CM were compared with 8 patients with CM of the medial femoral condyle. One patient with multiple localizations was excluded from this analysis. Subchondral bone layer was more frequently affected in the group with CM of the medial femoral condyle (ICRS score 3.75 ± 0.46 versus 3.17 ± 0.41, *P* = 0.0374), and KOSS score was higher in this group (8.12 ± 2.17 versus 5.5 ± 0.87, *P* = 0.0201). Although both parameters indicate the presence of more severe cartilage lesions in the group with CM of the medial femoral condyle, there were no further statistically significant differences between both groups, especially with regard to immunohistological and OA scores. Data are summarized in Table 4.

## 4. Discussion

### 4.1. BMP and Receptor Expression

The potential role for BMP-7 in cartilage repair has been demonstrated in various in vitro studies [8, 11, 30], showing BMP-7 induced proanabolic activity and elevated production of ECM components. Encouraged by this success, several in vivo studies and trials in humans have been undertaken that have confirmed a crucial role for BMP-7 in cartilage metabolism and OA development. For example a recent gene-array analysis revealed that BMP-7 is involved in the regulation of numerous key cytokines responsible for cartilage matrix production and modulation, and other anabolic or catabolic pathways in cartilage homeostasis as TGF-*β*/BMPs, IGF, and VEGF [31]. Even a phase 1 safety and tolerability study of BMP-7 application in symptomatic knee OA was initiated, suggesting a symptom response to the BMP-7 treatment together with a lack of dose limiting toxicity [32]. The intra-articular concentrations of BMP-7 in knee joints vary depending on the degree of CM [16]. In addition, amounts of plasma BMP-7 seem to show a positive correlation with synovial fluid BMP-7 levels, approving the potential role in cartilage repair and OA development [33]. A central question of the introduced study was to gain data about the localization of the BMPs and their receptors comparing expression in synovia and cartilage close to cartilage repair zones. A previously published study has already shown that human articular chondrocytes express BMP-7 with distinct patterns and correlations to its pro-form [10]. This could be confirmed by our results demonstrating strong BMP-7 expression in normal cartilage and bordering repair regions. Furthermore, intense BMP-7 signals were found in the synovia. Besides BMP-7 other members of the TGF*β* superfamily as BMP-3, CDMP-1, and CDMP-2 were detected in all layers of normal articular cartilage with the strongest expression in chondrocytes of the transitional layer [34]. Similar to BMP-7 a strong body of evidence indicates a crucial role for BMP-2 in natural and surgically induced cartilage repair [15]. For example, it has been shown that exogenous BMP-2 dramatically improved the chondrogenic character of amplified knee articular chondrocytes over two passages, as assessed by type II procollagen expression and synthesis [35]. A supposed basis of surgically induced cartilage repair is the recruitment and chondrogenic differentiation of mesenchymal stem cells (MSCs). Several studies have recently shown that BMP-2 applied together with TGF*β*1 or IGF-1 was able to induce a stable chondrogenic phenotype in MSCs of different origins [4, 36]. Data of these studies together with the presented results, showing expression of BMP-2 in cartilage close to repair zones and in synovia of knees with circumscribed cartilage lesions, suggest a potential key role for BMP-2 in these regenerating processes. In a previously published study it could be shown that BMP-2 is more consistent expressed in knee joints with local CM compared to BMP-7 [15]. Furthermore, BMP-2- but not BMP-7 levels were associated with a better clinical outcome. Immunostainings now seemed to demonstrate an opposite result. But there are several things to take into account. At first it has to be considered that only the worst regions with the lowest intensity or quantity of expression were evaluated. This had to be done, because partially normal cartilage was debrided at the edge of the biopsy. Considering this, it may be concluded that BMP-2-expression was more dependent on cartilage differentiation than expression of BMP-7, because BMP-7 scores were equally high in all samples and BMP-2 scores varied. Similar to our results, showing BMPR-IA expression in cartilage of human knees with focal CM, another study confirmed BMPR-IA expression in both normal and osteoarthritic articular cartilage. Data for BMPR-IB were also comparable demonstrating the lack of staining in OA cartilage [34]. This is slightly different to a study in rabbits; here BMPR-1B displayed the strongest staining of the BMP-receptors in both cartilage and bone, but BMPR-1A was also expressed in normal cartilage but not in calcified layers [9]. Taken together, all data suggest that not only BMP-2 or BMP-7 are expressed in cartilage but also their receptors with different intensity and quantity dependent on the species, and the different antibodies or staining protocols. Although there are some studies suggesting that BMP-2 significantly promoted the TGF*β*-induced chondrogenic differentiation of synovium-derived stem cells in vitro [37], data about expression of BMPs and their receptors in the synovia of knees with focal CM are still missing. Our results indicate that concentrations of BMP-2, BMP-7, and their receptors BMPR-1A, -1B, and -2 are similar in synovia and cartilage. This association is supported by experimental data gained during research in rheumatoid arthritis (RA). BMP signaling ligands were determined in synovium and cartilage extracts of arthritic knees with comparable activity, showing also downregulation of BMP-7 by inflammation-induced TNF*α* [38].

### 4.2. MRI, Clinical Parameters and Histology

MRI in association with quantitative image analysis enables the acquisition of quantitative information on articular cartilage physiology, pathophysiology and degenerative changes in OA [39, 40]. Immunohistological assessment of the expression of BMPs and their receptors was expected to be similarly associated with OA development; however, our data clearly show that a statistical correlation of histological data and quantitative radiography is missing. Apparent reasons are the focal nature of CM present in the evaluated population, and the weak association of BMP expression with the overall changes in the knee joint during OA development. KOSS score statistically significant correlated with duration of symptoms, which probably simply documents the progression of OA associated joint changes with time. This is also mirrored by the phenomenon of a better clinical outcome following microfracture treatment for focal cartilage lesions in patients with a history of knee complains less than 12 months before treatment [41].

### 4.3. Localization and Duration

A previously published review summarized the factors possibly influencing the clinical outcome after cartilage regeneration by microfracturing. Although it could be shown that age <40 years, duration of symptoms <12 months, lesion size <4 cm^2^, body mass index <30 kg/m^2^, preoperative Tegner score (activity level) >4, and no previous surgery seemed to positively influence the outcome, the authors were not able to evaluate the effect of localization of chondromalacia [42]. Other studies suggested that the best results in treatment of circumscribed cartilage lesions are found in young patients with defects on the femoral condyles [29]. These data let us evaluate the possible influence of defect localization on BMP expression. The subchondral bone layer was more frequently affected in the group with CM of the medial femoral condyle, and KOSS score was higher in this group. Although both parameters indicate the presence of more severe cartilage lesions in the group with CM of the medial femoral condyle, there were no further statistically significant differences between both groups, especially with regard to immunohistological and OA scores.

### 4.4. Conclusion

Summarizing, BMP-7 was consistently expressed in cartilage and synovial biopsies of patients undergoing ACI because of circumscribed cartilage lesions. BMPR-1A and BMP-2 were also found in a significant number of cases, but data indicate a stronger modulation of expression by the degree of CM. Although duration of symptoms statistically significant correlated with KOSS score, describing progress of OA in MRI by quantitative imaging, there was no influence of this parameter on protein expression. Although several scores indicate a more severe degree of CM in defects of the medial condyles compared to retropatellar damages, localization did not influence immunohistologically quantitated expression of BMP-2, BMP-7, and their receptors.

## Figures and Tables

**Table 1:** Specification of the used antibodies for immunohistology.

Antibody	BMP-2	BMP-7	BMPR-1A	BMPR-1B	BMPR-2
Specification	Mouse, monoclonal	Rabbit, polyclonal	Rabbit, polyclonal	Mouse, monoclonal	Rabbit, polyclonal
Dilution	1 : 200	1 : 200	1 : 25	1 : 200	1 : 50
Procedure for protein unmasking	Proteinase K (Dako REAL Proteinase K, Hamburg, Germany)	Proteinase K (Dako REAL Proteinase K, Hamburg, Germany)	Heat-induced antigen unmasking	Heat-induced antigen unmasking	Proteinase K (Dako REAL Proteinase K, Hamburg, Germany)
Manufacturer and product number	Abcam, ab6285, Cambridge, UK	Abcam, ab56023, Cambridge, UK	Lifespan Biosciences, LS-C4217, Seattle, USA	Abcam, ab78417, Cambridge, UK	Sigma, HPA017385, St. Louis, USA

**Table 2:** Correlation matrix.

	Defect size	KOSS (MRI)	Henderson score	Kellgren-Lawrence score	Duration (months)	Krenn score
Defect size						

Corr. coefficient	1.0000	**0.6259**	0.1567	**0.4705**	0.1478	−0.2382
Valid cases	15	**15**	15	**15**	15	15
Significance (*P*)	0.0000	0.0063*	0.2886	0.0384*	0.2995	0.1963

KOSS (MRI)						

Corr. coefficient	**0.6259**	1.0000	**0.6133**	0.2468	**0.6272**	−0.1165
Valid cases	**15**	15	**15**	15	**15**	15
Significance (*P*)	0.0063*	0.0000	0.0075*	0.1876	0.0062*	0.3397

Henderson score						

Corr. coefficient	0.1567	**0.6133**	1.0000	−0.2089	0.3768	**−0.4605**
Valid cases	15	**15**	15	15	15	**15**
Significance (*P*)	0.2886	0.0075*	0.0000	0.2274	0.0831	0.0421*

Kellgren-Lawrence score						

Corr. coefficient	**0.4705**	0.2468	−0.2089	1.0000	−0.3197	0.0330
Valid cases	**15**	15	15	15	15	15
Significance (*P*)	0.0384*	0.1876	0.2274	0.0000	0.1227	0.4536

Duration of symptoms (months)						

Corr. coefficient	0.1478	**0.6272**	0.3768	−0.3197	1.0000	0.1238
Valid cases	15	**15**	15	15	15	15
Significance (*P*)	0.2995	0.0062*	0.0831	0.1227	0.0000	0.3301

Krenn score (synovitis)						

Corr. coefficient	−0.2382	−0.1165	**−0.4605**	0.0330	0.1238	1.0000
Valid cases	15	15	**15**	15	15	15
Significance (*P*)	0.1963	0.3397	0.0421*	0.4536	0.3301	0.0000

*P < 0.05, corr. coefficient: correlation coefficient (Spearman-Rho), Syn: synovia, C: cartilage.

**Table 3:** Comparison between patients with different durations of complains (≤48 months *n* = 7, >48 months *n* = 8).

Criterion	Duration of symptoms		Significance (*P*)
	≤48 months	>48 months	
Age	39.22 ± 12.39	27.78 ± 6.732	0.0638*
Gender (f/m)	3/8	3/1	0.7552^#^
ICRS score	3.43 ± 0.53	3.62 ± 0.52	0.4623*
Defect size	3.68 ± 1.34	3.97 ± 1.67	0.4437*
KOSS (MRI)	5.86 ± 1.57	8.25 ± 1.98	0.0202*
Henderson score	2.00 ± 0.82	2.87 ± 1.36	0.1697*
Kellgren-Lawrence score	1.00 ± 0.58	0.87 ± 0.83	0.7023*
Duration of symptoms (months)	23.43 ± 16.36	76.50 ± 23.95	0.0010*
Krenn score	2.00 ± 1.41	2.25 ± 1.03	0.5048*
BMP-2-Syn	2.28 ± 1.89	2.37 ± 2.06	1.0000*
BMP-2-C	2.43 ± 1.90	2.25 ± 1.58	0.9500*
BMPR-1A-Syn	5.00 ± 1.00	4.75 ± 0.89	0.6171*
BMPR-1A-C	2.57 ± 1.62	2.62 ± 2.37	0.9000*
Mankin score	8.00 ± 2.00	7.375 ± 3.02	0.8590*
Pritzker score	3.43 ± 0.79	3.62 ± 1.51	0.9497*

*U-Test (Mann-Whitney), ^#^Fisher exact test, f: female, m: male, Syn: synovia, C: cartilage.

**Table 4:** Comparison between patients with different localizations of chondromalacia (retropatellar *n* = 6 or medial femoral condyle *n* = 8).

Criterion	Medial femoral condyle	Retropatellar	Significance (*P*)
Age	30.73 ± 10.85	35.49 ± 12.64	0.6982*
Gender (f/m)	2/3	3/5	0.9999^#^
ICRS score	3.75 ± 0.46	3.17 ± 0.41	0.0374*
Defect size	4.09 ± 1.79	3.29 ± 0.94	0.1882*
KOSS (MRI)	8.12 ± 2.17	5.5 ± 0.87	0.0201*
Henderson score	2.87 ± 1.36	2.00 ± 0.89	0.1826*
Kellgren-Lawrence score	0.75 ± 0.89	1.00 ± 0.00	0.3838*
Duration of symptoms (months)	65.25 ± 37.84	32.33 ± 21.55	0.0515*
Krenn score	2.25 ± 1.28	2.00 ± 1.26	0.6836*
BMP-2-Syn	2.62 ± 2.26	2.17 ± 1.60	0.9427*
BMP-2-C	2.00 ± 1.19	2.33 ± 2.06	1.000*
BMPR-1A-Syn	5.25 ± 0.89	4.50 ± 0.84	0.1279*
BMPR-1A-C	2.50 ± 2.26	2.50 ± 1.76	0.8860*
Mankin score	8.00 ± 3.34	7.67 ± 0.82	0.8943*
Pritzker score	4.00 ± 1.41	3.17 ± 0.41	0.2810*

*U-Test (Mann-Whitney), ^#^Fisher exact test, f: female, m: male, Syn: synovia, C: cartilage.

## Abbreviations

ACI: Autologous chondrocyte implantation
bFGF: Basic fibroblast growth factor
BMP: Bone morphogenetic proteins
BMPR: Bone morphogenetic protein receptor
CM: Chondromalacia
ECM: Extracellular matrix
ICRS: International cartilage repair society
IGF-I: Insulin-like growth factor-I
KOSS: Knee osteoarthritis scoring system
MSC: Mesenchymal stem cell
MRI: Magnetic resonance imaging
OA: Osteoarthritis
OP-1: Osteogenic protein-1
TGF-*β*: Transforming growth factor-*β*
JS: Joint space.
